# Plant regeneration via direct somatic embryogenesis from leaf explants of *Tolumnia* Louise Elmore ‘Elsa’

**DOI:** 10.1186/s40529-018-0220-3

**Published:** 2018-01-22

**Authors:** Hui-Ju Shen, Jen-Tsung Chen, Hsiao-Hang Chung, Wei-Chin Chang

**Affiliations:** 1 Institute of Plant and Microbial Biology, Academia Sinica, Taipei, 115 Taiwan, ROC; 2 Department of Life Sciences, National University of Kaohsiung, Kaohsiung, 811 Taiwan, ROC; 3 Department of Horticulture, National Ilan University, Yilan, 260 Taiwan, ROC

**Keywords:** Cytokinin, Direct somatic embryogenesis, Leaf explant, Light regime

## Abstract

**Background:**

*Tolumnia* genus (equitant *Oncidium*) is a group of small orchids with vivid flower color. Thousands of hybrids have been registered on Royal Horticulture Society and showed great potential for ornamental plant market. The aim of this study is to establish an efficient method for in vitro propagation.

**Results:**

Leaf explants taken from in vitro-grown plants were used to induce direct somatic embryogenesis on a modified 1/2 MS medium supplemented with five kinds of cytokinins, 2iP, BA, kinetin, TDZ and zeatin at 0.3, 1 and 3 mg l^−1^ in darkness. TDZ at 3 mg l^−1^ gave the highest percentage of explants with somatic globular embryos after 90 days of culture. It was found that 2,4-D and light regime highly retarded direct somatic embryogenesis and showed 95–100% of explant browning. Histological observations revealed that the leaf cells divided into meristematic cells firstly, followed by somatic proembryos, and then somatic globular embryos. Eventually, somatic embryos developed a bipolar structure with the shoot apical meristem and the root meristem. Scanning electron microscopy observations showed that the direct somatic embryogenesis from leaf explants was asynchronously. The somatic embryos were found on the leaf tip, the adaxial surface and also the mesophyll through a cleft, and it reflected the heterogeneity of the explant. The 90-day-old globular embryos were detached from the parent explants and transferred onto a hormone-free 1/2 MS medium in light condition for about 1 month to obtain 1-cm-height plantlets. After another 3 months for growth, the plantlets were potted with Sphagnum moss and were acclimatized in a shaded greenhouse. After 1 month of culture, the survival rate was 100%.

**Conclusions:**

In this report, a protocol for efficient regenerating a *Tolumnia* orchid, Louise Elmore ‘Elsa’, was established via direct somatic embryogenesis and might reveal an alternative approach for mass propagation of *Tolumnia* genus in orchid industry.

## Background

*Tolumnia*, previously referred to as “the equitant oncidiums”, is small and epiphytic with unapparent pseudobulbs that usually covered by leaves. The leaves arranged in pairs overlapping or straddling one another at the base. In cross section, the leaves showed circular or triangular shape. The inflorescence of *Tolumnia* orchids bears attractive and colorful flowers with large labella, and therefore became a popular pot plants in flower market worldwide. Based on our knowledge, there was no reliable protocol had been published for propagating *Tolumnia* in vitro.

In recent years, plant regeneration via direct somatic embryogenesis had been successfully established in various orchids including *Dendrobium* (Chung et al. 2005), *Epipactis* (Moradi et al. 2017), *Malaxis* (Mahendran and Bai 2016), *Phalaenopsis* (Chen and Chang 2006; Gow et al. 2010; Feng and Chen 2014), *Rhynchostylis* (Islam and Bhattacharjee 2015) and also a *Tolumnia* related genus, *Oncidium* (Chen et al. 1999; Chen and Chang 2002; Su et al. 2006; Hong et al. 2008a, b). In these previous studies, cytokinins, especially thidiazuron, were found to be effective to induce direct somatic embryogenesis from the leaf explants (Chen et al. 1999; Chen and Chang 2002; Su et al. 2006; Hong et al. 2008a, b). Therefore, to establish an efficient method for regenerating *Tolumnia* orchids, leaf explants taken from a popular cultivar, Louise Elmore ‘Elsa’, was used to test the effects of cytokinins on direct somatic embryo formation.

## Methods

### Plant materials

In vitro grown plants of *Tolumnia* Louise Elmore ‘Elsa’ were purchased from Taida Horticultural Co. Ltd., Chunghua County, Taiwan. These plants were maintained on a hormone-free 1/2 MS (Murashige and Skoog 1962) medium with sucrose (20 g l^−1^) and Gelrite (2.4 g l^−1^) at pH 5.2 in 250 ml flasks and subcultured every 2 months period. All of the cultures were incubated under a 16/8-h (light/dark) photoperiod at irradiance of 28–36 μmol m^−2^s^−1^ (daylight fluorescent tubes FL-30D/29, 40 W, China Electric Co., Taipei, Taiwan) and temperature of 26 ± 1 °C. Plants with several roots and leaves (about 2–4 cm in height) were used as donor plants.

### Embryo induction

The basal medium for embryo induction is a modified MS medium containing 1/2-strength macro- and full-strength micro-elements of MS salts supplemented with [mg l^−1^]: myo-inositol (100), niacin (0.5), pyridoxine HCl (0.5), thiamine HCl (0.1), glycine (2.0), peptone (1000), NaH_2_PO_4_ (170), sucrose (20,000) and Gelrite (2400). The pH of the media was adjusted to 5.2 with 1 M KOH or HCl prior to autoclaving at 121 °C for 15 min. One-year-old in vitro grown plants of *Tolumnia* Louise Elmore ‘Elsa’ were utilized as donor plantlets. Leaf tip segments (about 10 mm in length) excised from the top first leaves of donor plantlets were used to induce direct somatic embryogenesis on different media supplemented with five cytokinins, 2ip, BA, kinetin, zeatin and thidiazuron (TDZ) at 0, 0.3, 1 or 3 mg l^−1^, 2,4-D (0, 1, 3 mg l^−1^) combined with TDZ (0, 0.3, 1, 3 mg l^−1^). The leaf explants were placed on the culture and incubated in 90 × 15 mm^2^ Petri dishes under darkness or a light condition at irradiance of 28–36 μmol m^−2^s^−1^ as mention above in an incubator at temperature of 26 ± 1 °C.

### Plantlet development

Plantlets derived from somatic embryos with 3–4 leaves were transferred onto a hormone-free 1/2 MS medium with sucrose (20 g l^−1^) and Gelrite (2.4 g l^−1^) in 250 ml flasks with a 1-months-interval subculture period. All of the cultures were incubated under a light condition with 16/8-h (light/dark) photoperiod at irradiance of 28–36 μmol m^−2^s^−1^ and temperature of 26 ± 1 °C for further development. After 2 months of culture, the plantlets were transferred into plastic pots with Sphagnum moss in a shaded greenhouse for the further growth.

### Histological analysis

The tissue samples for histological observations were fixed in FAA (95% ethyl alcohol:glacial acetic acid:formaldehyde:water, 10:1:2:7) for 48 h and dehydrated in a tertiary-butyl-alcohol series (Liao and Wu 2011). Tissue samples were then infiltrated by liquid paraffin in the oven at 65 °C and embedded in paraffin wax. The wax blocks containing tissue samples were sectioned to a thickness of 10 µm using a Leica RM2125 RTS rotary microtome (Leica Microsystems) and stained with 0.5% safranin-O (Sigma-Aldrich Inc., USA) followed by 0.1% fast green (Sigma-Aldrich Inc., USA) (Jensen 1962). After staining, sections were permanently mounted on slides for observation.

### Scanning electron microscopy (SEM) observations

Samples for SEM were fixed in 2.5% glutaraldehyde in 0.1 M phosphate buffer (pH 7.0) for 4 h at 4 °C, and then dehydrated in ethanol (Dawes 1971), dried using a critical point dryer (HCP-2, Hitachi), and coated with gold in an ion coater (IB-2, Giko Engineering Co.). A SEM (DSM-950, Carl Zeiss) was used for examination and photography of the specimens.

### Data analysis

All the experiments were designed with a randomized complete block design. The percentages of explants forming somatic proembryos (smaller than 0.5 mm in diameter) and somatic globular embryos (larger than 0.5 mm in diameter) were counted under a stereomicroscope (SZH, Olympus, Tokyo, Japan). Data were scored after 75 and 90 days of culture. Five replicates (dishes) and each with four leaf explants were provided for each treatment. The data expressed as percentages were transformed using arc sine prior to ANOVA and then converted back to the original scale (Compton 1994). All treatment means were compared by following Duncan’s Multiple Range Test (Duncan 1955). Significant differences between means were presented at the level of p ≤ 0.05.

## Results and discussion

### The developmental pathway of direct somatic embryogenesis

**Fig. 1:**
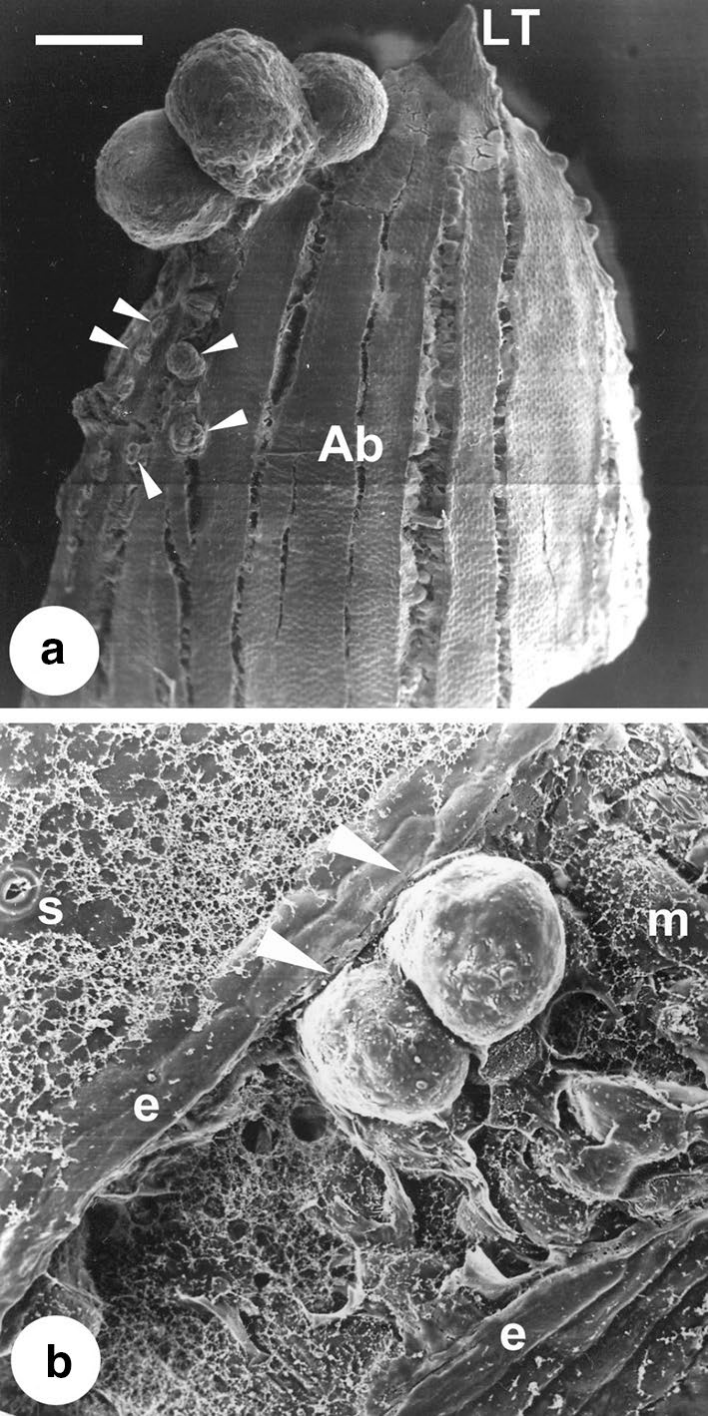
Scanning electron microscopic observations on direct somatic embryogenesis from leaf explants of *Tolumnia* Louise Elmore ‘Elsa’. **a** somatic proembryos (SPE, < 0.5 mm in diameter) and globular embryos (SGE, > 0.5 mm in diameter) formed from the leaf cells (*Scale bar* 1.5 mm); **b** a SPEs formed from the mesophyll cells (*Scale bar* 100 μm)

**Fig. 2:**
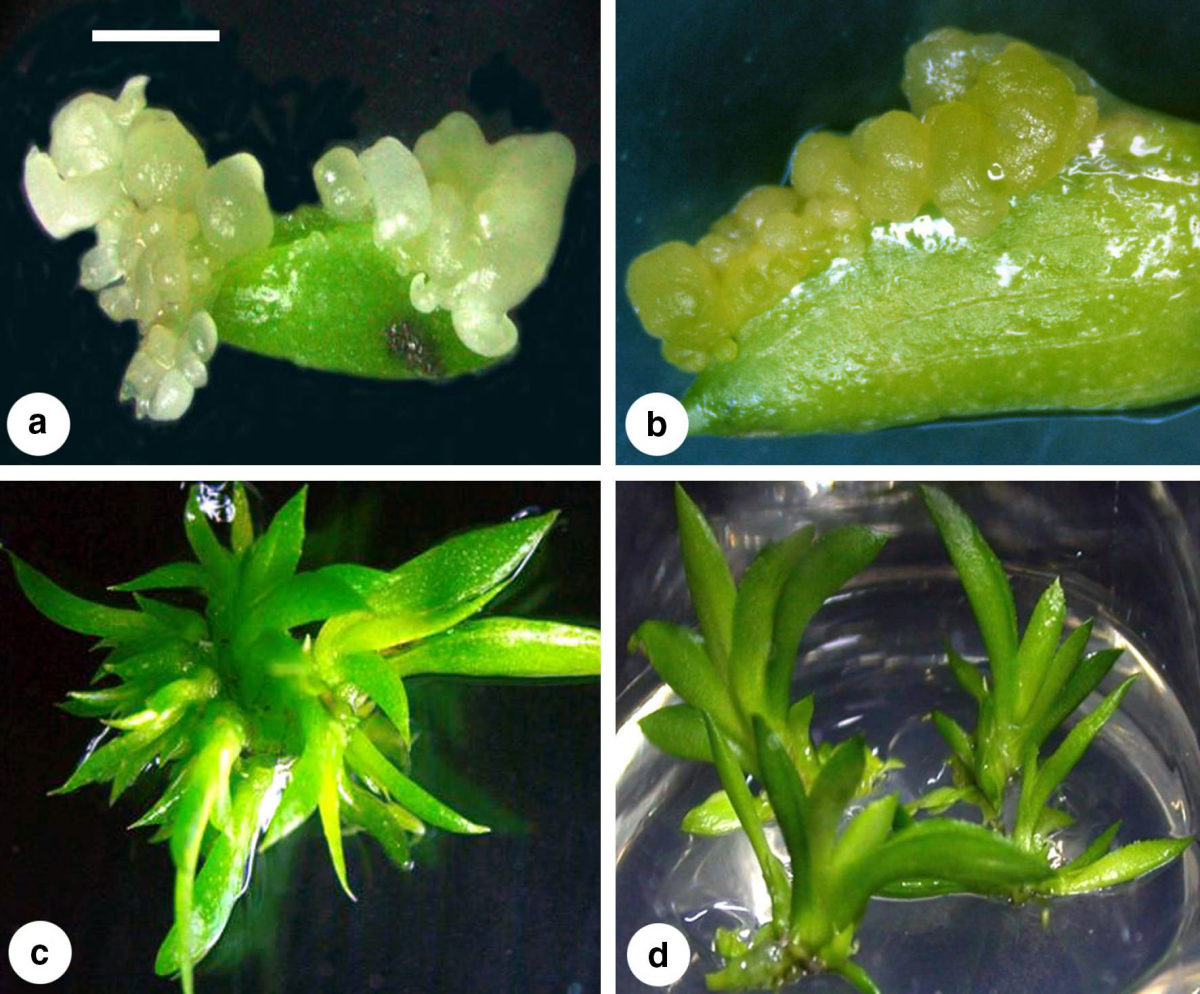
Somatic embryo development and plant regeneration from leaf explants of *Tolumnia* Louise Elmore ‘Elsa’. **a** somatic embryos formed on the leaf tip and the cut end of a explant (*Scale bar* 3.5 mm); **b** somatic embryos formed on the edge of the adaxial surface of the leaf explant (*Scale bar* 2 mm); **c** multiple shoots developed from leaf-derived somatic embryos (*Scale bar* 5 mm); **d** plantlets converted from leaf-derived somatic embryos (*Scale bar* 2.5 cm)

When the leaf explants were cultured on 1/2 MS medium supplemented with suitable concentrations of cytokinins (the details will be discussed in the following paragraph) for 60 days in darkness, transparent somatic proembryos directly formed from the epidermal cells (Fig. 1a) or from the mesophyll cells through a cleft (Fig. 1b). The SEM observations revealed that the size of these somatic proembryos was less than 0.5 mm in diameter (Fig. 1a, b). After another 15–30 days of culture on the same medium in darkness, some of the somatic proembryos developed into somatic globular embryos that showed whitish to light yellowish in color (Fig. 2a, b). Most of the somatic embryos were found on the leaf tips and the cut ends (Fig. 2a), but less than 10% of the explants (data not shown) formed somatic embryos from the adaxial sides (Fig. 2b). In general, the size of somatic globular embryos was about 0.5–2 mm in diameter (Fig. 1a).

### Somatic embryo development and plantlet establishment

The 90-day-old somatic globular embryos could be easily detached from the donor leaf explants and transferred onto hormone-free 1/2 MS medium in light condition. After 1–2 weeks of culture, the somatic embryos developed shoots and subsequent turned into 1-cm-height plantlets for another 2 weeks (Fig. 2c). After another 3 months of culture in light condition, morphological normal plantlets were ready to be transplanted into pots with Sphagnum moss (Fig. 2d). Dozens of plantlets were acclimatization in a shaded greenhouse for 1 month, and the survival percentage was 100% (data not shown).

### Histology of somatic embryo development

**Fig. 3:**
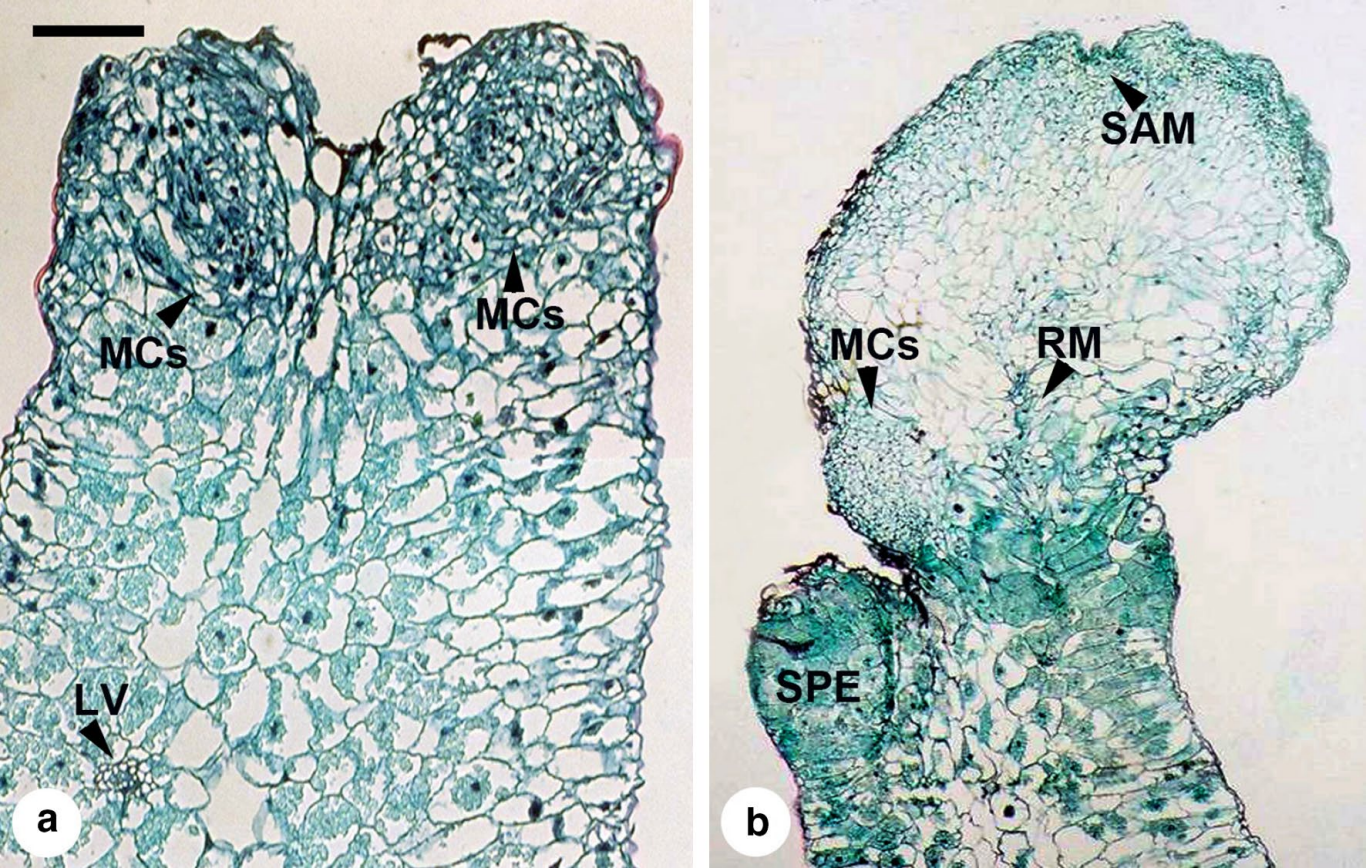
Histology of direct somatic embryogenesis from leaf explants of *Tolumnia* Louise Elmore ‘Elsa’. **a** meristematic cells (MCs) originated from the mesophyll cells of a leaf explant (*Scale bar* 0.1 mm); leaf vein (LV); **b** a somatic proembryo (SPE) and a somatic embryo developed the bipolar structure with putative shoot apical meristem (SAM) and putative root meristem (RM) (*Scale bar* 0.5 mm)

The somatic embryos were originated from the meristematic cells of the leaf explants (Fig. 3a). When compared to the original leaf cells, the meristematic cells showed smaller size and obviously densely stained (Fig. 3a). These meristematic cells were divided from the epidermal layers or together with the subepidermal layers of the explants (Fig. 3a). The somatic proembryo developed the typical bipolar structure with the putative shoot apical meristem and the putative root meristem (Fig. 3b). The somatic proembryo could be found on the same explant with the mature embryo, and thus the direct somatic embryogenesis showed an asynchronous behavior (Fig. 3b). On the posterior side of the mature embryos, the cells developed into meristematic cells, and thus suggested that secondary somatic embryogenesis will be in progress (Fig. 3b).

### Effects of cytokinins on direct somatic embryogenesis

Different concentrations of five kinds of cytokinins, 2iP, BA, kinetin, TDZ and zeatin, were tested for their effects on direct somatic embryogenesis (Table 1). After 75 days of culture in darkness, 2iP at 1 and 3 mg l^−1^, BA at 1 and 3 mg l^−1^, TDZ at 0.3, 1 and 3 mg l^−1^, zeatin at 0.3, 1 and 3 mg l^−1^ induced the leaf explants to form somatic proembryos (Table 1). The best result was found at 1 mg l^−1^ zeatin and 65% of the leaf explants were induced to form somatic proembryos in this treatment (Table 1). In the presence of TDZ at 0.3, 1 and 3 mg l^−1^, the somatic globular embryos were obtained from the leaf explants (Table 1). Other cytokinins were not able to induce the somatic globular embryos after 75 days of culture in darkness (Table 1). It may partially due to the explant browning that delayed or inhibited the transition from somatic proembryos to globular embryos (Table 1). High browning percentage (75–100%) of explants were obtained at the hormone-free control treatment, 0.3, 1 and 3 mg l^−1^ 2iP and 0.3, 1 and 3 mg l^−1^ kinetin, and it reflected the relatively lower percentages of somatic embryogenesis from the explants (Table 1). Therefore, these treatments were not suitable for inducing direct somatic embryogenesis (Table 1).

**Table 1:** Effects of cytokinins on direct somatic embryogenesis from leaf explants of *Tolumnia* Louise Elmore ‘Elsa’, when the explants were cultured in darkness for 75 days

Cytokinins	Concentration (mg l^−1^)	% of explants with somatic proembryos	% of explants with somatic globular embryos	% of browning
Hormone-free control	0	0 f	0 a	85.0 abc
2iP	0.3	0 f	0 a	90.0 abc
	1	20.0 def	0 a	75.0 c
	3	5.0 ef	0 a	80.0 bc
BA	0.3	0 f	0 a	30.0 d
	1	35.0 abcd	0 a	20.0 de
	3	15.0 def	0 a	10.0 de
Kinetin	0.3	0 f	0 a	100.0 a
	1	0 f	0 a	80.0 bc
	3	0 f	0 a	95.0 ab
TDZ	0.3	50.0 abc	5.0 a	5.0 de
	1	45.0 abc	10.0 a	0 e
	3	30.0 bcde	5.0 a	10.0 de
Zeatin	0.3	55.0 ab	0 a	10.0 de
	1	65.0 a	0 a	10.0 de
	3	30.0 cde	0 a	5.0 de

Means within a column followed by the same letter are not significantly different according Duncan’s multiple range test at p ≤ 0.05 (Duncan 1955)

After 90 days of culture in darkness, TDZ at 1 and 3 mg l^−1^, zeatin at 0.3, 1 and 3 mg l^−1^ induced significantly higher percentages of the leaf explants with somatic proembryos, and TDZ at 3 mg l^−1^ gave the significantly highest percentages of the leaf explants with somatic globular embryos (Table 2). In the presence of TDZ and zeatin, the browning percentages of explants were lower than 20%, and it reflected the relatively higher percentages of somatic embryogenesis from the explants (Table 2). In a previous report on *Oncidium* ‘Gower Ramsey’, a close relative species to *Tolumnia*, 0.3, 1 and 3 mg l^−1^ kinetin induced 40% of the leaf explants to form somatic embryos (Chen and Chang 2001). However, in this present study on the *Tolumnia* leaf culture, the result showed that kinetin could not induce the direct embryogenesis (Table 2). In the previous report, 1 and 3 mg l^−1^ TDZ induced 75% of the leaf explants to form somatic embryos after 40 days of culture in darkness, and it seems these embryos had no boundary to get maturity (Chen and Chang 2001). In this study, although higher percentages of explants with somatic proembryos (75 and 90%) were induced by 1 and 3 mg l^−1^ TDZ after 90 days of culture in darkness (Table 2). However, only lower percentages (20 and 35%, respectively) of explants with somatic proembryos got through into somatic globular embryos (Table 2). It was suggested that the timing and requirements of growth regulators on embryo development was vary between the orchid species.

**Table 2:** Effects of cytokinins on direct somatic embryogenesis from leaf explants of *Tolumnia* Louise Elmore ‘Elsa’, when the explants were cultured in darkness for 90 days

Cytokinins	Concentration (mg l^−1^)	% of explants with somatic proembryos	% of explants with somatic globular embryos	% of browning
Hormone-free control	0	0 d	0 d	100.0 a
2iP	0.3	0 d	0 d	95.0 ab
	1.0	20.0 cd	0 d	85.0 ab
	3.0	5.0 d	0 d	85.0 ab
BA	0.3	5.0 d	0 d	70.0 b
	1.0	60.0 ab	10.0 cd	35.0 c
	3.0	30.0 bc	5.0 cd	10.0 cd
Kinetin	0.3	0 d	0 d	100.0 a
	1.0	0 d	0 d	90.0 ab
	3.0	0 d	0 d	95.0 ab
TDZ	0.3	42.5 ab	15.0 bcd	5.0 d
	1.0	75.0 a	20.0 abc	20.0 cd
	3.0	90.0 a	35.0 a	15.0 cd
Zeatin	0.3	70.0 a	5.0 cd	10.0 cd
	1.0	90.0 a	20.0 abc	10.0 cd
	3.0	70.0 a	30.0 ab	10.0 cd

Means within a column followed by the same letter are not significantly different according Duncan’s multiple range test at p ≤ 0.05 (Duncan 1955)

### Effects of combinations of 2,4-D and TDZ on direct somatic embryogenesis

In previous reports, when 2,4-D combined with TDZ at appropriate concentrations, it was effective on embryogenic callus induction in *Oncidium* (Chen et al. 1999; Chen and Chang 2000). However, 2,4-D showed highly inhibitory on direct somatic embryogenesis in *Oncidium* (Chen et al. 1999; Chen and Chang 2001), *Dendrobium* (Chung et al. 2005, 2007) and *Phalaenopsis* (Kuo et al. 2005; Gow et al. 2008). In this present study, we combined 1 mg l^−1^ 2,4-D with 0, 0.3 and 3 mg l^−1^ TDZ to test the effect on direct somatic embryogenesis. Table 3 showed that at 1 mg l^−1^ 2,4-D plus 0.3 mg l^−1^ TDZ and 1 mg l^−1^ 2,4-D plus 3 mg l^−1^ TDZ, 5 and 10% of explants formed somatic proembryos, respectively. However, no globular embryos were found at these two treatments and it may due to the 100% of browning percentage (Table 3). In addition, the leaf explants did not form callus at all of the treatments.

**Table 3:** Effects of 2,4-D plus TDZ on direct somatic embryogenesis from leaf explants of *Tolumnia* Louise Elmore ‘Elsa’, when the explants were cultured in darkness for 90 days

2,4-D (mg l^−1^)	TDZ (mg l^−1^)	% of explants with somatic proembryos	% of explants with somatic globular embryos	% of browning
0	0	0 a	0	100.0 a
1	0	0 a	0	100.0 a
1	0.3	5.0 a	0	100.0 a
1	3	10.0 a	0	100.0 a

Means within a column followed by the same letter are not significantly different according Duncan’s multiple range test at p ≤ 0.05 (Duncan 1955)

### Effect of light regime on direct somatic embryogenesis

In previous reports, light regime was a negative factor that highly retarded the embryogenic response of leaf explants in *Oncidium*, *Dendrobium* and *Phalaenopsis* (Chen et al. 1999; Chung et al. 2005, 2007; Gow et al. 2009). However, in the root-derived callus culture of *Oncidium* ‘Gower Ramsey’, light regime was a crucial factor for inducing of indirect embryogenesis (Chen and Chang 2000; Wu et al. 2004). In this study, Table 4 showed that light regime highly retarded direct somatic embryogenesis and resulted in 95–100% of explants browning.

**Table 4:** Effects of TDZ and zeatin on direct somatic embryogenesis from leaf explants of *Tolumnia* Louise Elmore ‘Elsa’, when the explants were cultured in light condition for 90 days

Cytokinins	Concentration (mg l^−1^)	% of explants with somatic proembryos	% of explants with somatic globular embryos	% of browning
Hormone-free control	0	0 c	0	100.0 a
TDZ	1	10.0 abc	0	100.0 a
	3	5.0 bc	0	100.0 a
Zeatin	1	25.0 ab	0	100.0 a
	3	40.0 a	0	95.0 a

Means within a column followed by the same letter are not significantly different according Duncan’s multiple range test at p ≤ 0.05 (Duncan 1955)

## Conclusion

This present study is the first to establish an efficient protocol for regenerating a *Tolumnia* orchid via direct somatic embryogenesis. It took about 4 months to obtain the regenerated plantlets with normal morphogenesis. Plant regeneration was derived from leaf cultures via an in vitro morphogenetic pathway that meristematic cells developed initially, and followed by somatic proembryos, somatic globular embryos, somatic embryos with the bipolar structure, and eventually plantlets. This protocol provided the basis for further investigation on micropropagation, in vitro morphogenesis, gene transfer or breeding programs in *Tolumnia* orchids.

## Abbreviations

2,4-D: 2,4-dichlorophenoxyacetic acid
2iP: *N*^6^-2-isopentenyl adenine
BA: *N*^6^-benzyladenine
Kinetin: *N*^6^-furfuryladenine
TDZ: 1-phenyl-3-(1,2,3-thiadiazol-5-yl)-urea
Zeatin: 6-[4-hydroxy-3-methylbut-2-enylamino]purine
